# A Survey on Data-Driven Predictive Maintenance for the Railway Industry

**DOI:** 10.3390/s21175739

**Published:** 2021-08-26

**Authors:** Narjes Davari, Bruno Veloso, Gustavo de Assis Costa, Pedro Mota Pereira, Rita P. Ribeiro, João Gama

**Affiliations:** 1 Institute for Systems and Computer Engineering, Technology and Science, 4200-465 Porto, Portugal; narjes.davari@inesctec.pt (N.D.); bruno.m.veloso@inesctec.pt (B.V.); 2 Faculty of Science and Technology, University Portucalense, 4200-072 Porto, Portugal; 3 School of Economics, University of Porto, 4099-002 Porto, Portugal; 4 Federal Institute of Goiás, Campus Jataí, Unity Flamboyant, Jataí 75801-326, Brazil; gustavo.costa@ifg.edu.br; 5 Metro of Porto, 4350-158 Porto, Portugal; pm.pereira.mail@gmail.com; 6 Faculty of Sciences, University of Porto, 4169-007 Porto, Portugal

**Keywords:** condition-based maintenance, predictive maintenance, machine learning, deep learning, artificial intelligence, railway industry

## Abstract

In the last few years, many works have addressed Predictive Maintenance (PdM) by the use of Machine Learning (ML) and Deep Learning (DL) solutions, especially the latter. The monitoring and logging of industrial equipment events, like temporal behavior and fault events—anomaly detection in time-series—can be obtained from records generated by sensors installed in different parts of an industrial plant. However, such progress is incipient because we still have many challenges, and the performance of applications depends on the appropriate choice of the method. This article presents a survey of existing ML and DL techniques for handling PdM in the railway industry. This survey discusses the main approaches for this specific application within a taxonomy defined by the type of task, employed methods, metrics of evaluation, the specific equipment or process, and datasets. Lastly, we conclude and outline some suggestions for future research.

## 1. Introduction

Cyber-physical systems in Industry 4.0 are reforming conventional decision-making processes, mainly through the integration of entities and functionalities via intercommunication systems and intelligent data processing approaches. This reformation brings new challenges and high complexity. Operational decisions are tougher to be made. However, these advancements might provide new solutions for typical problems, as system failures, and thus, for maintenance approaches. Among many existing maintenance approaches, Predictive Maintenance (PdM) is a data-based approach that emerged as a prominent field of research. It uses statistical analysis, Machine Learning (ML) models, and Deep Learning (DL) solutions for modeling system behavior, discovering the trends and predicting failures, which improves a system’s reliability. PdM methods divide into three main categories, namely [1]: model-based prognosis, knowledge-based prognosis, and data-driven prognosis. Data-driven PdM strategies appeared with great prominence and importance both in industry and academia.

Detecting and preventing failures in industries with high operational risk (e.g., the railway industry) is ultimately essential to improve not only the system efficiency (e.g., equipment utilization) but also its effectiveness (e.g., the integrity of the environment and human safety). An effective maintenance management approach is vital, and industries seek to minimize the number of operational failures, minimize their operational costs, and increase their productivity. Consequently, planning and analysis strategies are necessary to assess the equipment’s operating status and useful life. However, due to the complexity involved in an industrial process, several automated solutions were implemented to perform future projections about the state of equipment by signal processing techniques that can support decision making.

This literature survey attempts to present, classify, and analyze the existing data-driven approaches developed for the PdM, specifically in the railway industry. Modern transportation is highly dependent on it to move cargo and passengers. The global increase in production and logistics needs higher use of the railway industry. Thus, common damages will occur in the overall structure and components due to factors such as weather and degradation. These could potentially lead to accidents of different proportions, which can even cause fatalities [2]. Indeed, operational and technical failures have a significant impact on the railway industry.

Recent advances in sensing and computing technology have given rise to PdM which, unlike traditional maintenance management techniques (e.g., corrective maintenance and preventive maintenance), attempts to predict failures and avoid system shut down proactively. Doing so maximizes system utilization, minimizes maintenance costs, and improves the system’s safety, reliability, and efficiency. Precisely, for the railway industry, with recent technology advances in cloud storage, communication, and sensing, we can monitor any part of the system more precisely and in real-time. Thus, it is a natural need for more complex solutions to analyze data with more scalability, precision, and efficiency.

In the past decade, a large number of works addressed PdM by the use of ML/DL approaches, but mainly the latter. The monitoring and logging of industrial equipment events, like temporal behavior and fault events, can be obtained from data and records generated by various sensors installed on the equipment. Specifically, sensors can be implemented to PdM in order to decrease the failure rate and enhance the system reliability [3]. Such sensors can monitor and generate alerts for equipment with the need for attention. Progressive development of industrial (wireless) sensor networks and emerging technologies, e.g., IoT [3,4,5], brings about generating a massive amount of data with scale and higher reliability. In this perspective, ML/DL algorithms are particularly relevant to create advanced mining methods for the PdM.

Research in PdM practices for the railway industry progressively receive more attention by the industry and academia. A recent literature review regarding Big Data Analytics in the railway industry can be found in [6], where the level and the types of big data models are reviewed and summarized for operations, maintenance, and safety applications. Most of the works focus on solutions that assess the infrastructure health state like railway points (switches) and interlocking systems. Although, in the case of trains, there exist many other challenges related both to internal conditions, like the general functioning of wagons (e.g., wheels, air compressed units, brakes) and external conditions, like weather, geographical position, in addition to other variables.

The dynamic context of the railway system is exceptionally challenging and these areas, by themselves, require the study of many combinations of analysis. In this sense, we define a taxonomy specific to the context of the railway industry. Differently, from [6], our taxonomy classifies the related works in three areas: infrastructure, scheduling policies, and vehicles. We also classify the works based on the type of data analysis method used to address PdM practices. We also employed a classification grounded on ML and DL algorithms, following the work in [1]. In practice, PdM needs a timely decision-making process which in turn needs models able to process data and adjust themselves in a timely manner.

In short, in this survey, we try to answer the following questions.

1. What parts of the overall railway industry are subject to PdM techniques?
2. What kind of data are being used with PdM?
3. How the DL methods are employed in the PdM applications?
4. What solutions are supported by DL methods and which are being used to perform PdM on the railway industry?

The contributions of this paper are threefold: (i) we review the maintenance applications, specifically the PdM practices describing the taxonomy of the solution space in addition to some technical aspects and current trends, (ii) we review recent advancements for data-driven PdM practices, specifically for the railway industry, and (iii) we present some of the main evaluation metrics for the PdM practices.

This paper is organized as follows. Section 2 presents and classify the PdM practices. Section 3 reviews the main ML and DL algorithms implemented for the PdM practices, also, the reader can find some of the most used datasets for Data-driven PdM, serving as a starting point for new projects. Section 4 specifically devoted to data-driven PdM practices in the railway industry, and Section 5 reviews the evaluation metrics for the PdM methods. Finally, in Section 6, we conclude with our final remarks and envision potential future research directions.

## 2. Predictive Maintenance

Maintenance corresponds to the process that deals with equipment or system components to ensure their normal functioning under any circumstances. Over the years, several different maintenance approaches have been developed, each representing a different generation over time due to technological advances. Three main maintenance approaches can be classified as below [7]:

- Corrective maintenance: it means run-to-failure, which is the simplest and the oldest method. The idea is to take action only after a machine or equipment fails. It would almost always lead to high (unexpected) downtime, besides having maintenance staff expenditure. This method usually generates a critical situation that will demand a great cost for companies.
- Preventive maintenance: it provides planning of regular replacement of components and/or equipment. Considering historical failure data and/or the data provided by the equipment manufacturer, MTTF is calculated, which in turn is used by the maintenance team to propose a preventive action plan. Although this approach prevents unexpected shutdown, it usually needs additional costs and an increased unexploited lifetime.
- PdM: it needs direct monitoring of the mechanical condition and other parameters that can determine the operating conditions over time. Indeed, due to technological advances, existing tools can process real-time data acquired from different equipment parts to predict any sign of failure.

**Figure 1:**
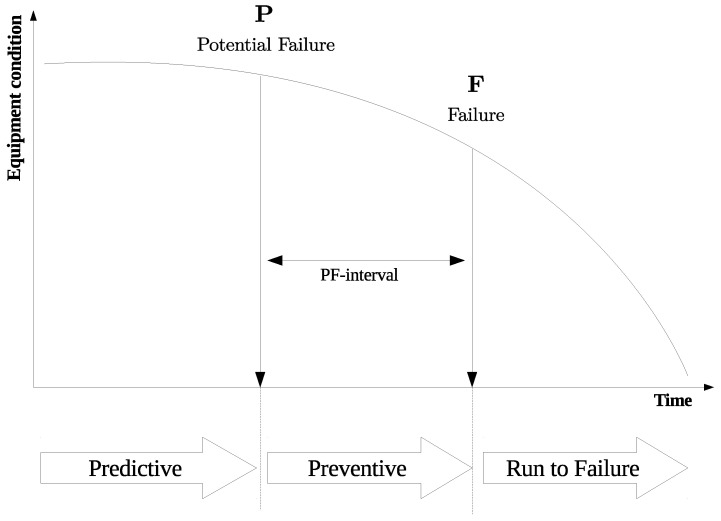
P-F reliability curve in maintenance management [8].

An equipment failure is almost random and unpredictable which is impacted by several (unknown) factors. A well-known technique to decide on the maintenance approach is P-F curve analysis (cf. Figure 1), which allows understanding the condition of equipment over time [8]. During the time between the detection of potential failure and the actual failure, it is crucial to perform a maintenance action to address the problem before a functional failure occurs.

The improvement of computing capacity, communication, and storage infrastructure allowed the triggering of PdM of mechanical equipment as the focus of the next stage of development [5]. In industrial manufacturing, IoT embedded in machines and production lines is now a reality. Large-scale stream processing for real-time data also becomes a reality that needs to be considered by industries, mainly because of competitive issues. PdM became one of the central answers to this challenge [9].

The most common data collected from sensors are vibration, thermography, and tribology [7]. PdM planning usually uses data streams to obtain operational conditions information and predicts equipment failures. Usually, it contributes to cost reduction and the overall improvement of quality in production. Nevertheless, results could still be better if we make use of data from more sensors or even the combination of some of them [8].

**Figure 2:**
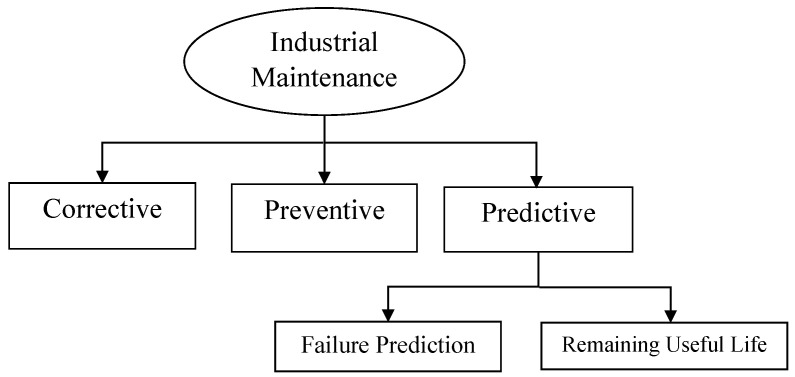
Classification of automatic industrial maintenance approaches.

Over the years, PdM practices have been developed from several perspectives; namely, ref. [10]: (i) f6+ailure prediction, to predict equipment failure overtime interval; (ii) RUL estimation, to estimate the remaining useful lifetime of equipment. These two perspectives are illustrated in Figure 2 and are detailed next.

### 2.1. Failure Prediction

Failure Prediction is the most generic and direct perspective for the PdM practices for which the main goal is to predict the approximate moment where some failure could occur.

PdM is generally employed based on the health status of critical elements. In an attempt to avoid possible interruptions or even more severe damage, based on the operational history of different components, this strategy can be used to predict failures over time, minimizing costs and extending the useful life of the components.

### 2.2. Remaining Useful Life (RUL)

Different maintenance management policies can be employed by the use of anomaly detection, diagnostics, and prognostics [11]. The RUL is strongly related to prognostics, which provides the amount of time equipment will be operational before it requires any repair or replacement. Prognostic is directly related to MTTF estimation and the likelihood of system failure occurrence. It can be regarded as a forecasting process given the current machine conditions and its historical record [12].

Based on the application type, goals may differ, i.e., PdM can be performed to predict the RUL of a specific asset or a set of assets to predict failure within a given time window or even just flagging abnormal behavior in a system. Current works reflect this modeling behavior, as will be seen in the following sections.

A categorization of methods and techniques for RUL can be found in [13]. As a fundamental task for RUL, prediction clearly defines the difference between run-to-failure (corrective maintenance) and time-to-failure (prognostics) strategies.

## 3. Data-Driven PdM

Unlike the model-based maintenance approaches (e.g., preventive maintenance approaches) that rely on forecasting the performance degradation by the use of stochastic models, data-driven PdM practices are based on data without prior knowledge of degradation conditions. Its performance strictly depends on the analysis of signals and data. While for complex systems, model-based solutions can be expensive and inaccurate, data-driven diagnosis methods are a promising alternative to fault/anomaly detection and isolation [14]. ML and DL algorithms and tools are naturally relevant to the PdM practices, mainly due to a large amount of data (specifically the unlabeled ones). Based on the availability of data and respective labels, learning methods can be classified into three different categories: (i) supervised learning, in which a labeled training data set are used for a mapping from the set of predictor variables values to a specified target variable; (ii) semi-supervised learning, where the goal is to learn from data sets that have the target variable value for only a subset of examples [15]; and (iii) Unsupervised learning, in which machine learns from data sets with no target variable.

In addition, RL and DL are also mainly implemented often under the scope of semi-supervised and/or unsupervised approaches [16]. The former is a technique that looks forward to discovering the actions needed to maximize a numerical reward in a trial-and-error fashion, while the latter is defined by the structure and functions of NNs [17]. DL differs on how features are handled. There is a hierarchy with features at different levels, where the composition of low-level features forms higher-level ones and, complex functions can be learned by mapping the input to the output [18].

Recent reviews on the ML/DL methods for PdM are found in the literature. We highlight some of those next. In [16], authors describe the recent advances in techniques and applications. In [19] the authors provide a review of the recent advancements of ML/DL techniques applied to PdM for smart manufacturing, and the works are classified based on ML/DL algorithms, ML/DL category, machinery and equipment used, device used in data acquisition, and data size and type. Finally, in [20] authors provide an insight into ML/DL used for PdM practices and provides an overview of industrial sensors and future research aspects of sensors in PdM practices.

Regarding the data available for the PdM practices, it is challenging to assign labels to the real-time data stream from sensors in an industrial plant. Firstly because of the limited types of measurements and secondly because of the cost and feasibility of having one or more specialists analyze data. Thus, we can argue that using supervised learning is not a feasible solution way in this context. Another important aspect is the scale. Different types of sensors are massively being adopted for use in a great variety of automation applications. With the IoT paradigm, new challenges are imposed for the storage and retrieval of large amounts of data and their meaningful visualization [21].

The last 6 years have been very productive in PdM research and works with ML/DL methods for industrial applications are becoming the majority of them. The current advances in this area contribute mutually to enhancing methods and the improvement of industrial planning. From this scenario, we can conceive many challenges. Next, we review the main ML and DL tools implemented in PdM practices and on the following public datasets available on the Web for PdM is reviewed.

### 3.1. Traditional Machine Learning Methods

Several ML algorithms and methods have been used to predict failures and RUL. Some approaches explored the use of classical algorithms as LR [22], SVR [23], SVM [24], RF [25] while osthers explored the combined use of algorithms with step phased approaches: ARIMA and SVM [26], SVR and SVM [27] and TL with RF [28]; and also with a comparative approach: RF, QRF, DT, KNN, SVR and PCR [25]. In here, we briefly review recent works used traditional ML methods in PdM applications.

AE, a network trained to attempt to copy its input to its output, is widely used in PdM practices. It is a method well-suited for unsupervised feature extraction. Based on the AE architecture, many works have adopted a common solution of extracting features from the input in an attempt to reduce concerns of overfitting in the models [29,30,31,32,33,34,35,36], or as in the case of [37], where AE was used as part of the ensemble model.

To make simple AE more robust, a Variational AE (VAR) is also proposed for learning deep latent-variable models and corresponding inference models by the use of stochastic gradient descent. In [38], the Variational AE was used to deal with insufficient labels in an asset failure prediction application.

Baptista et al. [39] proposed a framework based on ARMA to make predictions as an alternative to traditional life usage modeling. The case study involved a critical component of commercial aircraft. Zheng [40] presented a method to predict a bearing RUL based on a health indicator algorithm and a linear degradation model. Ordóñez et al. [26] proposed an algorithm supported by ARIMA and SVM models for RUL prediction of aircraft engines.

Using Empirical Mode Decomposition and Wavelet Transforms as pre-processing techniques to improve input quality, coupled with Particle Swarm Optimized Support Vector Machines (PSO+SVM), Souto Maior et al. [41] has estimated the RUL of bearing from the IEEE PHM Challenge 2012 big dataset.

Zhang et al. [42] proposed to use transfer learning with bi-directional LSTM for RUL estimation. They firstly train the models on different but related datasets and then fine-tuned by the target dataset. The performance of the estimation model is evaluated with two measures that were used: Scoring Function [43] and RMSE.

### 3.2. Deep Learning Methods

Traditional ML approaches show better performance for lesser amounts of input data. However, advancements in sensing technologies and the emergence of technologies such as IoT produce a vast amount of data, and consequently, the performance of traditional ML techniques could not meet the required scale. In this context, DL becomes a necessary choice [16]. DL techniques process highly non-linear and varying sequential data with minimal human input in several knowledge domains [44].

A recent survey in [45] presents a systematic review specifically DL techniques applied to PdM practices, where the DL benefits and limitations for fault diagnosis and prognostics are discussed. Another recent review for DL techniques applied to PdM practices can be found in [46]. Nevertheless, another recent review can be read in [47] specifically for DL applied to machine health monitoring in which an overview on AE and its variants and RBM and its variants including DBN and DBM, CNN, RNN are presented.

In addition to the review works, some recent works proposed to perform a comparative analysis of their PdM strategy to different classical ML algorithms [48,49,50,51]. Given the steadily increasing use of sensors and the amount of data produced by them, and the fact that these data are often materialized as real-time time series DL methods will undoubtedly be among the future PdM tools. Thus, in the following subsections, we will give focus on DL algorithms and methods.

#### 3.2.1. Deep Neural Network (DNN)

A DNN is an ANN with multiple layers (more than two hidden layers) between the input and output layers without looping back, and the flow of the network goes through the layers, calculating the probability of each output [52,53].

Among the early applications of DL methods, we can refer to a multi-layer feed-forward ANN for engine fault diagnosis is developed in [54], an ANN method to classify diesel engine fault occurrences in [55], a feed-forward ANN prediction model to estimate conditions of laser welding processes in [56], and a two-layer ANN for a fault diagnosis framework which can learn features extracted from mechanical vibration signal.

Several relevant works also employed DNN to develop prediction models. In general, the goals are to diagnose different elements of an industrial plant, e.g., wind turbine gearbox [57], rolling bearings, and planetary gearboxes [58], among others [59,60,61,62,63].

#### 3.2.2. Convolutional Neural Network (CNN)

A CNN is a type of DNN that is trained with the backpropagation algorithm and is common in image processing tasks [64] and is widely used for PdM practices. A diagnosis strategy to detect the fault type in the planet bearing is proposed in [65]. The strategy is based on the SST, where the Hilbert transform processes raw vibration signals to obtain the fault information. The 1D time-series signals are converted into 2D images, from which a DCNN can automatically learn underlying fault features by fault classification. Additionally, DCNN used in [66] to monitor the wear condition of an abrasive belt from grinding sound signals. Another fault recognition method for rotating machinery is proposed in [67] in which a multi-sensor data fusion and bottleneck layer optimized CNN is used to (i) convert vibration signals from multiple sensors to 2D images and (ii) extract features and fuse the multi-sensor data.

Fault diagnosis is also considered in Chen et al. [68], where a CNN and DWT method is used to identify the fault conditions of planetary gearboxes of wind turbines. CNN is used to learn the discriminating features from the coefficients of DWT. Moreover, Ma and Chu [37] proposes a diagnosis method for rotor and rolling bearings faults based on an ensemble DL formulation, which in turn is based on a multi-objective optimization algorithm. The ensemble learning approach is based on ResCNN, DBN and Deep AE.

CNN methods are also used for RUL estimation; e.g., Wang et al. [10] proposes an approach supported by Functional Data Analysis (FDA) for RUL estimation. The method incorporates the correlations within the same equipment and the discrepancy across sensor time series from different equipment. Additionally, Al-Dulaimi et al. [69] propose a Hybrid DNN model for RUL estimation that integrates two parallel paths (one LSTM and one CNN) followed by a fully connected multilayer fusion NN which combines the output of the two paths to form the target RUL.

#### 3.2.3. Recurrent Neural Network (RNN)

In contrast to feed-forward networks, in RNN feedback loops are possible. Additionally, a cascade of neurons get fired in this kind of network, and the output of a neuron only affects its input at some later point in time, i.e., they have some limited duration before becoming inactive.

In [70], a method based on LSTM RNN, is proposed to assess bearing performance degradation. LSTM is an RNN architecture that has feedback connections and, in addition to single data points, it can also process sequences of data. A bearing degradation indicator is constructed to represent the bearing running states, validated with feature verification and selection by a simulation model based on a vibration response mechanism. Another LSTM architecture is proposed in [71] to predict whether a truck compressor failure will happen within a specified time window of 90 days. However, Nguyen and Medjaher [72] design a LSTM classifier to calculate the probabilities that the system will fall into different time intervals.

In [73], authors present two models to capture and encode characteristics of signals, or groups of signals on-board vehicles caused by air compressor faults in city buses. One approach used histograms, and the other is based on echo state networks (ESNs), a specific type of RNN, that exhibits fast training without local optima, and it is used for modeling the signal. Recently, Gugulothu et al. [74] present an approach based on RNN that processes sensor data in a sequence-to-sequence model to generate embeddings for multivariate time series. They generate separate embeddings for normal machines and degraded machines and, after comparison, it is possible to estimate the RUL, even in the presence of noise in sensor readings.

More recently, a RNN classifier has been introduced by Onchis [75] for condition monitoring of cantilever beams. They used the changes in natural frequencies based on time-frequency processing extracted from vibrating beams. Most recently, Lepenioti et al. [76] implements a RNN for predictive analytic and a multi-objective RL method for prescriptive analytic. The proposed method was implemented for a PdM scenario in a steel-making company.

#### 3.2.4. Generative Adversarial Network (GAN)

CAN is an approach to generative modeling using DL, where two NNs compete with each other. It offers an alternative approach to maximum likelihood estimation techniques [16]. Yoon et al. [38] present a semi-supervised learning approach for modeling failures when there is a lack of a high number of labels on historical data. Using a non-linear embedding technique, based on a variational AE, they combined a GAN model parameterized by DNN. Authors have also used turbofan engine degradation data sets from NASA CMAPSS [77].

In a recent work Shao et al. [78] propose the framework based on GAN) to learn from mechanical sensor data. The framework composes of two parts: generator and discriminator. The network makes use of stacking one-dimensional convolution layers to learn local features from the original input. Most recently, two GAN networks were proposed in [79] for failure prediction based on experimental data collected from an Air Pressure System (APS) data set [80] and a turbofan engine degradation data sets from NASA CMAPSS [77].

Finally, we summarize the works on general data-driven solutions for PdM in Table 1. This table is outlined by employed methods and data sources, the equipment or process where the solutions were applied, and the respective references. From Table 1 we can observe that independently of the Goal or the Learning Task, most used techniques rely on different types of neural networks, showing the applicability of these techniques on different data sources (type of sensors/equipment).

### 3.3. Datasets for PdM

Some public datasets for testing and evaluating PdM techniques in different scenarios are provided in [87]. PdM strategy is distinctive and application-dependent, supported by the environment, available data, hardware, among others. Thus, these data sources give support to the development, testing, and comparisons with different ML techniques.

For failure prediction methods, a dataset proposed by [88] for a robot failure can be used, in which 463 samples and 30 attributes are provided. A second data source, proposed by [89], aimed to detect faults and estimate weights for a gearbox using some data and information about bearing geometry. In the dataset in [90], component failures were detected in the air pressure system of trucks, from where 76,000 samples and 171 attributes were obtained. A fourth data set, proposed by [91] is composed of faults detected from robot swarms.

For the mechanical failures, a well-known dataset, the Commercial Modular Aero-Propulsion System Simulation (C-MAPSS) [77] developed by NASA to simulate the operation of turbofan engines. The Case Western Reserve University Bearing Data Center (CWRU) [92] contains motor bearing data from different operation condition, as normal operating state, single-point drive, and fan defects. The third dataset can be considered as the one proposed in the Numenta Anomaly Benchmark (NAB) [93], where NAB version 1.1 is composed of over 50 labeled real-world and artificial time series data files. Measurements from motor current and vibration signals from the Paderborn University bearing Dataset [94] enable the verification of models and sensors of different signals to increase the accuracy of fail detection from bearings. We also can introduce PRONOSTIA [95], a popular dataset for predicting bearing’s RUL. It is known as the bearing accelerated life test dataset, which serves to investigate new algorithms. It provides real data related to the accelerated degradation of bearings in different operating conditions.

In Table 2, we collected the datasets mentioned above that can support experiments and comparative analysis in PdM studies. For each dataset, we provide the reference and a brief description.

## 4. Data-Driven PdM for the Railway Industry

PdM practices in the railway industry are not so recent as with many other application areas. However, recent advancements of AI technologies provide new opportunities for its expansion. Although ML/DL methods developed for the PdM practices in a wide range of applications, the literature with specific applications in the railway industry is yet scarce. A recent review regarding the data-driven PdM works in the railway tracks can be found in [96]. The works have been classified based on model types and application types. Their study indicates that in the new research trend ML/DL methods, unsupervised methods, and ensemble methods are the most implemented learning methods. Next, we also provide a review of the works developed between 2000 and 2021, classified in infrastructure, scheduling policies, and vehicles topics.

### 4.1. Infrastructure

Automated inspections and maintenance prediction of the infrastructure is becoming a major concern for the rail industry practitioners. Examples include but are not limited to the works reported for rail tracks and anchors. Failures on railway tracks can cause many problems related to costs, and consequently, there is great demand imposed to maintain rail tracks in a good state of repair [82].

Among the first works, an SVM based algorithm to predict impending failures and alarms of critical rail car components is proposed in [97], in which they use data from sensors installed along the railway. Recently, a data-driven PdM method has been developed in [98] for the railroad switch which is an arrangement of equipment that enables railway trains to switch from one track to another. Faults in this system can cause traffic delays. The author uses the data available from maintenance bookkeeping and railway controlling system logging. The proposal faced the problem with a supervised learning strategy to make predictions and tests are performed by SVM, RF, naive Bayes generative model, and LR methods. Railway tracks are critical components in the rail industry. Faults and failures will necessarily occur to tracks as with any other mechanical system with time and usage.

Another recent work in [99] proposes tree-based classification techniques (e.g., decision tree, random forest, and gradient boosted trees) for the maintenance need prediction, activity type, and trigger’s status of railway switches. This study criticized the expensiveness of employing additional data collection measures to record the assets’ behavior. The author has utilized historical data of visual inspection, condition state, and maintenance records. From comprehensive maintenance action data, e.g., visual inspections and maintenance records, this classification technique employs multiple models based on a DT, an RF and GBT.

More recently, ref. [100] design a four-layer big data architecture for establishing a data management framework to manage enormous amounts of data produced by railway switch points. A LSTM prediction model is implemented within the framework for detecting failures based on analytical tasks in the Italian railway industry. Additionally, a data-driven risk prediction model to predict and evaluate rail defects and service failures is proposed in [101], in which a framework to predict the risk of rail defects recurrence in different segments of the network is also developed.

Lately, an advanced data mining method based on ML techniques to create strategic decision support and draw up a risk and control plan for trains was proposed in [102]. They used stored-inactive data from a Greek railway company for the random forest classifier and decision tree classifier algorithms trained by the historical data for 6 years. According to the experience extraction from domain experts and the available resources from the system, the approach improves operations efficiency.

### 4.2. Scheduling Policies

Recent reviews for the railway industry [82,103] reveals that most works address track defects using corrective maintenance. In addition, the scheduling process is mainly planned in cases when defects are already known. Among the few works considered data-driven PdM practices, predictive and risk-based maintenance activities schedule is considered in [104], in which predictions for maintenance of railway infrastructure are performed by predicting the degradation state of certain assets. A two-stage stochastic linear program forecasts the future track conditions.

A data-driven policy for the inspection and maintenance of track geometry to give support on both corrective and preventive maintenance is proposed in [82], where a Markov chain and Bernoulli process were used to modeling data from some observed magnitudes. The results using RF, SVM and LR algorithms are compared and further used to model the relationship between the explanatory and the dependent variables. Moreover, a MCMC simulation is employed to calculate and compare the total cost of different policies.

An integrated method for the prediction of rail and geometry defects and optimal scheduling is proposed in [105]. In railway industry terminology, geometry defects are horizontal and/or vertical misalignment on the track, while rail defects include track wear such as corrosion or impairments such as broken rails or cracks. The solutions provide inspection and maintenance schedules. The authors make use of K-means to perform feature selection, followed by predicting the number of defects by RF and RNN methods. Moreover, a MDP to integrate the stochastic nature of defect occurrence into scheduling is used to find the optimum inspection policies.

### 4.3. Vehicles

Considering the components for which a data-driven PdM is practisced, vehicle maintenance prevails with a particular emphasis on the maintenance of four components: wheel, bearing, truck, and traction. In an early work, a knowledge discovery solution is presented to extract data from historical behavioral data collected by sensors in [106]. It is based on association rules, more specifically sequential pattern mining, to extract specialized classes. Using anomaly detection, they compare new patterns with sequential patterns describing normal behavior that were extracted before. Later, a RF based methodology was developed in [25] to assess the current health and predict RUL of both trucks (bogies) and wheels of a rail-car by fusing measurements from three types of detector. The MissForest, an RF based non-parametric imputation method, is also used to handle missing data in detector reading. The work in Fumeo et al. [85] deals with data streams coming from onboard sensors to make RUL predictions. They proposed a novel algorithm based on Streaming Data Analysis (SDA), where predictions are performed with online-SVR.

Recently, data extraction from open/close cycles controlling valves of a train door is proposed in [2], where the authors aimed to detect structural failures in the train door controlling system. Firstly, an anomaly detection algorithm is used with the support of different windowing strategies. After that, a low-pass filter is applied to the output in an attempt to improve anomaly detection. In addition, a temporal factor is incorporated in both phases.

DNN and traditional data-driven methods, regarding the extraction of fault features, are compared in [107]. These features should represent, effectively, essential information aiming to perform an intelligent diagnosis. The fault signals of bogies with big data were processed using a DNN, and the corresponding results are compared with those from a multi-hidden layer neural network, a single hidden layer neural network with a shallow structure. The work concludes that DNN can improve identification accuracy and are extremely useful in reducing defects into manually designing the features. A framework to detect air leakage and predict its severity to determine action plans is presented in [22], in which anomalies are detected to find air leakages from the logs of a compressor. The method is based on a LR classifier to model different classes of compressor behavior for the trains from a fleet. It also employs a clustering method to differentiate anomalies from outliers. The author claims that most failures can be detected one to four weeks before the occurrence and that their contextual anomaly detection method can avoid false alarms. They made use of real datasets from Dutch Rail.

Most recently, an online detection model for train speed is proposed in [108], in which an anomaly detection strategy and a Bayesian statistical model that represents train behavior in speed changes are developed. A linear regression model is employed, taking into account the time duration and travel distance from the departure station. In this study, the OpenRails platform is used to simulate the operation of trains and generate data aiming to evaluate the performance of the model. A learning method for the prediction of wheelsets RUL and failure types, combining linear regression loss, LR loss, and L2/L1 regularization, is proposed in [27]. The method is based on SVM for failure type classification and SVR for RUL prediction.

### 4.4. Overview

Following the literature we reviewed in the previous sections, a summary is presented in Table 3. Generally, it is possible to verify that a significant part of the references was conducted by supervised learning. The exceptions are the works in [2,105], which make use of semi-supervised and unsupervised learning, respectively. Moreover, there is an almost exact division in task employment, i.e., half-used anomaly detection and other half used prediction.

Excepting the works in [22], and ref. [27] that propose to perform both Failure Prediction (FP) and RUL estimation, all the other works aimed to reach distinct goals. As can be observed from Table 3, only two papers addressed RUL estimates for some railway assets while the rest proposed to predict some type of failure.

As we stated before, supervised learning is not a feasible solution in the context of PdM for the railway industry because it makes predictions based on known training examples. In addition, as the operation of this system is dynamic over-functioning time, we can realize one first challenge of having a model that can be updated in real-time (online learning) for the anomaly detection task. There are several challenges in robustly learning the distribution for any time series without any supervision [109].

More than half of these works gave attention to the maintenance need of trains behavior in the sense of cost reduction and accident avoidance. In the current context, this attention will increase due to the new challenges involving new ways of measuring and detecting the different parts of the train system in a multivariate analysis fashion. Another important aspect is the data types used in the experiments. Most of them were real data extracted from sensors/monitors, as stated in [87].

## 5. Evaluation Metrics in PdM!

In this section, we provide a review of the metrics used for performance evaluation of the PdM practices, specifically in the railway industry. Reviews for the measurement of the performance of anomaly detection methods and prognostic systems can be found in [110,111]. The most common performance evaluation metrics in the context of PdM are reported in Table 4 and described next.

### 5.1. Failure Prediction

The metrics proposed for the performance evaluation of Failure prediction methods mainly measure the number of failures predicted accurately and/or the number of wrong predicted failures. Accuracy is a natural metric through which the number of true predicted failures and true predicted non-failures over a total number of events is measured. The performance of the DNN developed for fault prediction in bogies in [107] was evaluated through the Accuracy metric. It also has been used to evaluate the performance of the fusion algorithms based on neural networks proposed in [5] for mechanical fault diagnosis. Accuracy, misclassification rate, and f-score were also used in [99] to evaluate the performance of classification technique for maintenance prediction of railway switches.

The other principal evaluation metric is PR score, in which the percentage of truly identified failures over the number of predicted failures (true or false) is calculated (precision) and is compared to the percentage of the failures identified truly overall the failures (recall) [112]. PR score was used to evaluate the sensors data pattern mining approach developed in [106] and to evaluate the performance of fault prediction of railway track geometry developed in [82].

PR score has also been used to evaluate the failure prediction developed in [98] for data of maintenance bookkeeping and system logging. The authors also made use of AUC-ROC [114] to evaluate prediction performance and error analysis.

The performance of the integrated inspection and maintenance scheduling operations proposed in [105] for train geometry defects predictions were evaluated using MAE and RMSE metrics. RMSE was also used in [22] to evaluate a logistic regression classifier and a density-based clustering method proposed for anomaly detection. Moreover, the failure prediction method proposed in [39] based on operational log data was evaluated through Accuracy and precision were the metrics approached, in addition to RMSE, the median absolute deviation, and MTBF, a metric from the reliability domain.

In [2], authors adapted two metrics, namely: rFAR and rIPR, to deal with outlier detection, benefiting from the early failure detection. The rFAR reduces the number of false alarms, appearing just before the correct identification of a failure. In rIPR reduce the number of impostors for appearing after the correct identification of a failure.

### 5.2. Remaining Useful Life

MAE, MAPE, MSE, and RMSE are among the most common performance metrics used to evaluate RUL prediction methods. MAPE and MSE were used to evaluate the RF based methodology was developed in [25] to predict RUL of both trucks and wheels of a rail-car. The MAPE was also used for performance evaluation of RUL prediction proposed in [27], in which the authors also used PR for the classification result, and RUL estimation of bearings proposed in [85].

The MAE and MAPE were used to evaluate an approach for RUL estimation on two datasets was proposed and evaluated in [74], and an algorithm based on ARIMA and SVM proposed in [26] for RUL estimation. MAPE and RMSE were also used in [28] to evaluate a mapping function using RF regression model for predicting RUL of equipment under the scenario that labeled data are only available for the source domain.

The other performance metric includes confusion probability in [72] for an LSTM classifier proposed to perform prognostics and Accuracy in [113] for an approach for RUL estimation on two datasets was proposed and evaluated in [74].

## 6. Conclusions and Future Directions

In this survey, we reviewed the main works developed ML/DL algorithms for PdM in the railway industry. Some questions were initially outlined, but during the review, we also got an overview of new trends and challenges that can be faced by academia and industry.

Although the data-driven PdM are gaining more research attention, specifically in the past few years, the number of works specifically designed for the railway industry is quite limited. Initially, we were interested in the works including the vehicles, e.g., the general functioning of wagons. However, the limited number of works led us to consider a broader context.

Considering the research trends reviewed in the previous section, we can observe some significant gaps to be researched in future works. As noted, only a few works have faced the problem of using data as time series. Sensors typically gather data in the time-series format. Thus, we can envision this scenario as a task of anomaly detection in time series. Anomaly detection is the problem characterized by identifying specific patterns or events in data that are pretty different from the rest. Anomalies can arise in the data for many reasons, and one of the most common examples is malicious activities, as in the case of credit card fraud.

In manufacturing systems, reducing downtime is critical, and anomaly detection enables PdM for downtime reduction. Recent works have addressed anomaly detection for PdM supported by learning strategies on sequential data [2,39,106,115,116,117,118]. In the last few years, several papers were published approaching Anomaly Detection with Time-Series data applied to the most different domains, including industry, public water, and energy systems, among many others [1,109,112,114,118,119,120,121,122,123,124,125,126,127,128,129,130,131,132,133,134,135,136,137,138,139,140].

Dealing with models high volume of time-series in real-time to perform anomaly prediction is the major challenge. Moreover, currently used metrics are not feasible in this context, and it will be indispensable to look for new alternatives that can efficiently evaluate models.

The other essential line of action is to look for different DL algorithms and architectures like RNN, GAN, TL and RL. Recent works have proposed approaches based on DL to resolve the problem of anomaly detection in time-series [28,125,127,139,141,142]. Nevertheless, new proposals in this research line will be necessary.

The last challenge would be to achieve the desired synergy between ML/DL methods and RCA by gaining automatic reasoning power to explain causality, which these methods by themselves are unable to perform.

## Figures and Tables

**Table 1:** ML/DL methods used for PdM.

Goal	Learning Task	ML/DL Method	Data Source	Equipment/Process	Ref.
Failure Prediction (FP)	Anomaly Detection	Hierarchical Clustering	General faults	Time and Frequency	[81]
	Classification	RF, SVM and LR	Physical faults	Track geometry	[82]
		AE	General faults	Rolling bearing	[35,36]
				Spacecraft	[32]
				Transformers	[34]
				Rotor bearing systems	[37]
			Vibration	Tidal turbine	[33]
				Bearings	[29]
			Acoustic signals Sensor data	Motors	[83]
		DNN	Vibration	Bearings	[84]
				Gasoline engines	[54]
				Engines	[63]
			Vibration, pressure and speed	Diesel engines	[55]
			Optical and visual	Laser welding	[56]
		CNN	Vibration	Planetary gearbox	[65,68]
			Grinding faults	Abrasive belt wear	[66]
			General faults	Rotor bearing systems	[37]
			Vibration and images	Rotating machinery	[67]
		RNN	General faults	Rolling bearing	[70]
				Air compressor	[71]
				Air compressor in buses	[73]
			Sensor data	Turbofan engine degradation	[72]
			Time-frequencies	Cantilever beams	[75]
		GAN	Sensor data	Turbofan engine degradation	[79]
			Vibration	Induction motor	[78]
Remaining Useful Life (RUL)	Regression	Online-SVR	Vibration	Rolling Bearing	[85]
		PSO+SVM	Vibration	Rolling Bearing	[41]
		Bi-directional LSTM	Sensor data	Turbofan engine degradation	[42]
		AE	Acoustic signal Sensor data	Turbofan engine degradation	[38]
		CNN	Sensor data	Turbofan engine degradation	[10,69,86]
		RNN	Sensor data	Turbofan engine degradation	[74]

**Table 2:** List of datasets publicly available for PdM experiments.

Ref.	Dataset Description
[93]	Numenta Anomaly Benchmark (NAB) dataset: temperature sensors on industrial machines
[88]	Force and torque measurements to detect robot failures
[89]	Failure data of a generic gearbox
[92]	CWRU: ball bearing test data for normal and faulty bearings
[94]	Synchronous measurement of motor current and vibration signals
[90]	Operational data from a pressurizing system in trucks
[95]	PRONOSTIA: bearing accelerated life test dataset
[77]	NASA C-MAPSS tools: simulate realistic large commercial turbofan engines
[91]	Failure data in a simulated swarm of robots

**Table 3:** Data-driven PdM for the railway industry.

Goal	Learning Task	ML/DL Method	Data Source	Equipment/Process	Ref.
Failure Prediction (FP)	Anomaly Detection	Sequential Pattern Mining	Real Data: sensors on trains	Trains	[106]
		AE, **OCC!**, **OCSVM!**, boxplotEns	Real Data: sensors on trains	Pneumatic valves of train doors	[2]
		Linear Regression	Openrails simulation platform	Train speed	[108]
	Classification	SVM	Real Data: detectors on the railway	Railway	[97]
		LR, Bayes Classifier, SVM, RF	Real Data: log files and reports	Railway turnouts	[98]
		RF	Public Real Data	Railway track geometry	[82]
		ANN	Software SIMPACK	Trains	[107]
		RF, RNN, K-means	Real defect database	Rail and geometry defects	[105]
		DT, RF, Gradient Boosting Trees	Real Data: SAP/ERP Maintenance Request Process (MRP)	Railway switches	[99]
Remaining Useful Life (RUL)	Regression	online-SVR	Real Data: detectors on trains	Train axle bearings	[85]
		RF, QRF, DT, KNN, SVR, PCR	Real Data: detectors on trains	Wheels and trucks (bogies)	[25]
		SVR, SVM	Real Data: North America Railroad	Train wheelsets	[27]
		LR	Real Data: Dutch Railways VIRM	Air leakage in braking pipes of trains	[22]

**Table 4:** Evaluation Metrics used in PdM.

Metric	Ref.
Accuracy	[5,82,98,99,105,107]
PR	[27,82,106,112]
Confusion Probability Matrix	[72,99]
RMSE	[22,25,26,28,39,74,105,113]
MAE	[26,74,113]
MAPE	[25,27,28,74,85,113]
AUC-ROC	[98,114]
rFAR and rIPR	[2]

## Data Availability

Not applicable.

## Abbreviations

The following abbreviations are used in this manuscript:

AE	Auto-Encoder
AE	Auto-Encoder
AI	Artificial Intelligence
ANN	Artificial Neural Network
ARMA	Auto-regressive Moving Average
ARIMA	Auto-Regressive Integrated Moving Average
AUC-ROC	Area Under Curve/Receiver Operating Characteristic
BN	Bayesian Network
CNN	Convolutional Neural Network
DBN	Deep Belief Network
DBM	Deep Boltzmann Machines
DCNN	Deep Convolutional Neural Network
DL	Deep Learning
DRL	Deep Reinforcement Learning
DNN	Deep Neural Networks
DT	Decision Tree
DWT	Discrete Wavelet Transformation
GAN	Generative Adversarial Network
GBT	Gradient Boosted Tree
GRU	Gated Recurrent Units
IoT	Internet of Things
KNN	K-Nearest Neighbour
LSTM	Long Short-Term Memory Network
LR	Logistic Regression
MAE	Mean Absolute Error
MAPE	Mean Absolute Percentage Error
MCMC	Markov Chain Monte Carlo
MDP	Markov Decision Process
ML	Machine Learning
MLP	Multi-Layer Perceptron
MSE	Mean Squared Error
MTBF	Mean Time Between Failure
MTTF	Mean Time To Failure
NN	Neural Network
PCR	Principal Component Regression
PhM	Prognostic and Health Management
PdM	Predictive Maintenance
PR	Precision-Recall
QRF	Quantile Regression Forests
ResCNN	Residual Convolutional Neural Network
RBM	Restricted Boltzmann Machines
RCA	Root Cause Analysis
RL	Reinforcement Learning
RF	Random Forest
rFAR	reduced False Alarm Rate
rIPR	reduced Impostor Pass Rate
RMSE	Root Mean Squared Error
RNN	Recurrent Neural Network
RUL	Remaining Useful Life
SCM	Structural Causal Models
SST	Synchro-Squeezing Transform
SVM	Support Vector Machine
SVR	Support Vector Regression
TL	Transfer Learning
